# Allopregnanolone and progesterone in relation to a single electroconvulsive therapy seizure and subsequent clinical outcome: an observational cohort study

**DOI:** 10.1186/s12888-024-06167-3

**Published:** 2024-10-15

**Authors:** Elin Thörnblom, Janet L. Cunningham, Malin Gingnell, Mikael Landén, Jonas Bergquist, Robert Bodén

**Affiliations:** 1 Department of Medical Sciences, Uppsala University, Entrance 10, Uppsala, 751 85 Sweden; 2 Institute of Neuroscience and Physiology, Sahlgrenska Academy at University of Gothenburg, Gothenburg, Sweden; 3 Department of Medical Epidemiology and Biostatistics, Karolinska Institute, Stockholm, Sweden; 4 Department of Chemistry – BMC, Analytical Chemistry and Neurochemistry, Uppsala University, Uppsala, Sweden

**Keywords:** Electroconvulsive therapy; epileptic seizures, Allopregnanolone, Progesterone

## Abstract

**Background:**

Electroconvulsive therapy (ECT) is an important treatment for several severe psychiatric conditions, yet its precise mechanism of action remains unknown. Increased inhibition in the brain after ECT seizures, mediated by γ-aminobutyric acid (GABA), has been linked to clinical effectiveness. Case series on epileptic patients report a postictal serum concentration increase of the GABA_A_ receptor agonist allopregnanolone. Serum allopregnanolone remains unchanged after a full ECT series, but possible transient effects directly after a single ECT seizure remain unexplored. The primary aim was to measure serum concentrations of allopregnanolone and its substrate progesterone after one ECT seizure. Secondary aims were to examine whether concentrations at baseline, or postictal changes, either correlate with seizure generalization or predict clinical outcome ratings after ECT.

**Methods:**

A total of 130 participants (18–85 years) were included. Generalization parameters comprised peak ictal heart rate, electroencephalographic (EEG) seizure duration, and prolactin increase. Outcome measures were ratings of clinical global improvement, perceived health status and subjective memory impairment. Non-parametric tests were used for group comparisons and correlations. The prediction analyses were conducted with binary logistic and simple linear regression analyses.

**Results:**

Allopregnanolone and progesterone remained unchanged and correlated neither with seizure generalization nor with clinical outcome. In men (*n* = 50), progesterone increased and allopregnanolone change correlated negatively with EEG seizure duration. In a subgroup analysis (*n* = 62), higher baseline allopregnanolone and progesterone correlated with postictal EEG suppression.

**Conclusions:**

ECT seizures have different physiologic effects than generalized seizures in epilepsy. Progesterone might have implications for psychiatric illness in men.

## Background

Generalised epileptic seizures induced in electroconvulsive therapy (ECT) alleviates a wide range of serious conditions, such as severe depression and mania, acute psychosis, catatonia, postpartum psychosis, schizophrenia, status epilepticus and Parkinson’s disease, but its mechanism(s) of action remains elusive [1]. Deficient inhibitory function has been implied in several psychiatric illnesses, including affective disorder [2–5], schizophrenia [6, 7], and catatonia [8, 9]. Animal models of ECT have found postictal increase of the inhibitory neurotransmitter γ-aminobutyric acid (GABA) and GABAA-receptor upregulation [10–12], which mediated alleviation of induced depression-like states [13]. In humans, postictal electroencephalography (EEG) suppression, reflecting GABA-ergic inhibition [14], has been linked to ECT response [15–17].

The neuroactive steroid allopregnanolone (ALLO) is a positive modulator of the GABA_A_ receptor [18]. ALLO is a metabolite of progesterone (PROG), and both steroids are synthesized in the central nervous system as well as peripherally in the adrenal glands and gonads [18, 19]. Both molecules cross the blood-brain-barrier, but concentrations are approximately ten times higher in blood than cerebrospinal fluid, likely reflecting that adrenal and gonadal glands are the main source of peripheral ALLO and PROG [18–20]. In animal models and human subjects, circulating ALLO concentrations increase after acute stress, and the administration of ALLO and PROG mitigates the hypothalamic‒pituitary‒adrenal axis response to acute stress in rodents [21]. Altered peripheral ALLO concentrations have been reported in mood disorders, post-partum depression, and animal models of depression and anxiety [18]. ALLO deficiency contributes to aberrant GABA signalling in major depression and postpartum depression [21], the latter of which can be treated with both synthetic ALLO [22] and ECT [23]. Case reports on epileptic seizures have reported increased postictal serum concentrations of ALLO [24, 25] but not PROG [24]. As ALLO augments GABA signalling, increases in peripheral blood after epileptic seizures, and alleviates postpartum depression, it is possible that ALLO contributes to the postictal GABAergic activity of ECT. A previous study reported no change in ALLO or PROG plasma concentrations after a full series of ECT treatments [26]. However, potential changes might be transient, as reported in animal models of stress and case reports of patients with epilepsy [21, 24, 25]. To the best of our knowledge, possible transient increases in circulating ALLO and PROG directly after a single ECT seizure have not previously been explored.

If ALLO and PROG increase directly after an ECT seizure and correlate to clinical parameters used to measure ECT seizure generalization, this could imply involvement in postictal GABA signalling and affect treatment outcome even if no lasting changes can be detected at the end of an ECT series.

Furthermore, both ALLO and PROG increase the seizure threshold [27], but whether individual serum concentrations affect ECT seizure quality is currently unknown.

Another possible link between neuroactive steroids and the effects of ECT is hippocampal neurogenesis, as both ALLO and PROG stimulate dendrite formation and synapse formation in the hippocampus [28]. In rodents, electroconvulsive stimulation induces hippocampal neurogenesis, which seems to be linked to antidepressant-like effects [29]. Hippocampal neurogenesis promotes learning and the formation of new memories but can induce amnesia as new memories are formed [30]. Retrograde amnesia is a side effect of ECT, although improved cognitive functioning has also been reported, and findings vary between populations and methods of assessment [31, 32]. Clinical studies on the impact of ALLO and PROG on cognitive function have shown conflicting results [28, 33]. Thus, ALLO and PROG might contribute to both the antidepressant and cognitive effects of ECT.

## Methods

### Aims

The primary aim of this study is to test the hypothesis that serum concentrations of ALLO and its substrate PROG increase after a single ECT seizure. A second aim is to examine whether baseline concentrations or postictal changes in ALLO and PROG correlate with seizure generalization parameters. We hypothesized a negative correlation between baseline concentrations and markers of seizure generalization but a positive correlation between postictal concentration increase and markers of seizure generalization. The third and final aim is to explore whether baseline concentrations or postictal changes in ALLO and PROG predict clinical improvement or acute subjective memory impairment at the end of an ECT series.

### Study design and participants

This is an observational add-on cohort study at one site (Uppsala University Hospital) of the Swedish multicentre study Predictors For ECT (PREFECT) [34, 35] and shares some data with a previous report on clinical ECT seizure parameters [36]. The results were presented in poster and abstract form at the 2022 conference of the European College of Neuropsychopharmacology [37].

All patients referred for clinical ECT between January 2014 to June 2016 were invited for participation in the multicentre study if they were at least 18 years old, able to give written informed consent, and planned for a minimum of six ECT sessions. There were no further exclusion criteria. This study builds on blood samples drawn before and after the first ECT seizure of a treatment series as part of the original multicentre design. Three types of data were utilized: blood sampling, a retrospective chart review of treatment and seizure parameters at the local site, and rating scales from the Swedish National Quality Register for ECT (Q-ECT).

### ECT treatment

ECT treatments were given between 8 a.m. and 12 a.m., with a Thymatron^®^ System IV device (Somatics LLC, Lake Bluff, IL, USA). Only right unilateral electrode placement was used. The stimulus charge was determined by the ‘age method’ [38], and median charge was 251 with first and third quartiles (Q1, Q3) 152 and 350 mC, respectively. For anaesthesia, thiopental was used for 127 of 130 participants, with a median dose (Q1, Q3) of 4.43 (3.91, 4.90) mg/kg. For one participant, propofol (2.46 mg/kg) was used. For two participants, data on anaesthesia were missing.

### Outcome variables

The main outcome variables were serum concentrations of ALLO and PROG measured immediately before the first ECT treatment and within 30 min after the finished seizure. Secondary outcome variables were seizure generalization parameters and clinical outcome ratings. Generalization of the ECT seizures was evaluated using four parameters: peak heart rate (HR) during the seizure, EEG seizure duration, postictal EEG suppression, and postictal increase of serum prolactin concentration (Δ-prolactin). Clinical outcome after the completed ECT series were assessed with three rating scales: the Clinical Global Impression-Improvement Scale (CGI-I) [39] for clinical improvement, the self-reported Euro-Qol Visual Analogue Rating Scale (EQ-VAS) for perceived health status [40], and the memory item from the Comprehensive Psychopathological Rating Scale (CPRS-M) [41] for subjective memory.

### Methods of measurement and data requisition

Blood samples were drawn immediately before and within 30 min after the first ECT seizure in the series. The exact sampling time was not recorded. Blood samples were drawn in serum tubes, rested at room temperature for 30–60 min, centrifuged for 15 min at 2000×g, and stored at -20 °C for a maximum of 30 days before transfer to -70 °C.

Serum prolactin concentrations had been measured as part of the larger multicentre study using the proximity extension assay technique (Olink Proseek^®^ Multiplex ONC Lv2 panel, OLink Bioscience, Uppsala, Sweden) [42]. ALLO and PROG concentrations were measured with ultra-performance supercritical fluid chromatography-tandem mass spectrometry [43]. The level of detection, level of quantification, and coefficient of variance were 0.02 ng/mL, 0.05 ng/mL and 5.1%, respectively, for ALLO and 0.01 ng/mL, 0.05 ng/mL and 0.9%, respectively, for PROG. Twenty samples were below the level of quantification, for which the level of detection was registered as the sample concentration. Of these analyses, eleven were performed before the seizure (eight ALLO, three PROG), and nine were performed after the seizure (seven ALLO, two PROG). In another twelve samples, no analyte was detected, and 0 ng/mL was registered as the sample concentration. These were all ALLO analyses, six before the seizure and six after.

Peak HR, EEG seizure duration, and postictal EEG suppression (present or absent) were assessed by clinical staff as per clinical routines based on recommendations in the ECT Handbook by the Royal College, third edition [44]. Peak HR was assessed via continuous electrocardiography. EEG seizure duration and postictal EEG suppression were assessed via visual inspection of a 2-channel bilateral frontomastoid EEG recording.

Clinical staff rated improvement with CGI-I within one week after the last treatment of the full ECT series (number of treatments was determined by the referring physician and not regulated in the study protocol). Patients rated EQ-VAS and CPRS-M before starting ECT and within one week after the last treatment. All ratings were reported to the Q-ECT, from which study data were retrieved. The Q-ECT reported a national coverage rate of 85% in 2013, the year preceding this study [45]. The CGI-I is a physician-rated 7-point Likert scale for improvement, as follows: 1 = very much improved, 2 = much improved, 3 = minimally improved, 4 = no change, 5 = minimally worse, 6 = much worse, and 7 = very much worse [39]. Clinical improvement on the CGI-I was defined as 1–2 points. The EQ-VAS is a patient-rated visual analogue scale ranging from 0 (worst imaginable state of health) to 100 (best imaginable state of health) [46]. The CPRS-M is a patient-rated 7-point Likert scale, with definitions for every second step: 0 = no memory deficits, 2 = temporary memory impairment, 4 = troublesome or embarrassing memory impairment, and 6 = total inability to remember anything. Memory impairment on the CPRS-M was defined as an increase of ≥ 2 points after ECT.

### Statistical methods

Descriptive data were tabulated using means and standard deviation (SD) when normally distributed, and using medians with the first and third quartile (Q1 and Q3) when not normally distributed. The Mann-Whitney U test was used for comparisons of neuroactive steroid concentrations in men and women.

For the primary analysis, differences in ALLO and PROG concentrations before/after the ECT seizure were assessed using the sign test.

For the secondary analysis, correlations between neuroactive steroid concentrations and continuous ECT seizure generalization parameters (peak HR, EEG seizure duration, and Δ/baseline prolactin) were assessed with Spearman’s rank correlation test. Neuroactive steroid concentrations in seizures with/without postictal EEG suppression were compared with the Mann‒Whitney U test. Correlation analyses of concentration changes primarily used the variable Δ/baseline concentration to correct for possible floor or ceiling effects of baseline values. Statistical results were then confirmed by analysing correlations to unadjusted Δ-concentrations.

For the third analysis, potential effects of hormone concentrations on outcome ratings were explored with regression analyses. Ratings on the Likert scales CGI-I and CPRS-M were dichotomized. CGI-I ratings were grouped into either ‘clinical improvement’ (ratings of 1 or 2) or ‘no clinical improvement’ (ratings ≥ 3). CPRS-M ratings were grouped into either ‘memory impairment’ (increase of ≥ 2 points after the completed ECT series) or ‘no memory impairment’. The dichotomized outcomes were then entered into separate logistic binary regression models. The ordinal outcome EQ-VAS score was entered into a simple linear regression model. Baseline and Δ-concentrations of each hormone were then entered as separate predictors of the respective outcome.

The post-hoc analysis used the Mann-Whitney U test to compare baseline ALLO/PROG concentrations and differences between sexes, and the sign test to assess changes in ALLO/PROG after the seizure.

All analyses were performed with SPSS Statistics for Windows, versions 27.0 and 28.0 (IBM Corporation) with the significance level α < 0.05.

## Results

### Participants

Of the initial 133 participants with samples from the PREFECT biobank, 130 were included. Two participants were excluded because no retrospective chart review was possible. One participant was excluded since they had erroneously been included at the end of the ECT series. Age, seizure parameters and clinical outcome ratings are shown in Table 1. 

**Table 1:** Participant age, indication for ECT, seizure evaluation parameters, and outcome ratings

	Whole cohort (*n* = 130)	Women (*n* = 80)	Men (*n* = 50)
Age, years, median (Q1,Q3)	45 (30,58)	43 (33,57)	47 (30,65)
Depression, n	123	76	47
Bipolar depression, n	29	19	10
Psychotic depression, n	12	6	6
Other indication, n^a^	7	4	3
Δ/baseline-prolactin, NPX, median (Q1,Q3)^b^	1.13 (0.45,2.74)	1.46 (0.37,3.04)	1.00 (0.55,1.88)
Peak HR, bpm, mean (SD)^b^	120 (24)	121 (23)	120 (25)
EEG seizure duration, s, median (Q1,Q3)	40 (25,54)	40 (24,54)	40 (27,55)
Postictal EEG suppression, present/absent, n^b^	51/11	29/7	22/4
CGI-I, n (%)^b^			
Improved^c^	86 (74)	50 (68)	36 (84)
No change	30 (26)	23 (34)	7 (16)
Worsened	1 (1)	1 (1)	0 (-)
EQ-VAS, median (Q1, Q3)^b^			
Before ECT	30 (20, 40)	30 (20,40)	30 (20, 50)
After	60 (40, 75)	60 (37, 75)	60 (50, 78)
Change	25 (10, 41)	24 (10, 40)	25 (11, 44)
Δ-CPRS-M, n (%)^b^			
Improved^c^	19 (22)	10 (19)	9 (29)
No change	47 (55)	28 (52)	19 (61)
Worsened	19 (22)	16 (30)	3 (10)

^a^Other indications (ICD-10-SE code): 1 bipolar mixed episode (F31.6), 1 severe manic episode with psychosis (F31.2), 2 other bipolar disorder (F31.8), 1 bipolar disorder unspecified (F31.9), 1 acute and transient psychotic disorder (F23.9), 1 borderline personality disorder (F60.3).

^b^Missing data: 42 in Δ/baseline-prolactin, 6 in peak HR, 68 in EEG suppression, 13 in CGI-I, 24 in EQ-VAS before, 35 in EQ-VAS after, 49 in Δ-EQ-VAS, 45 in Δ-CPRS-M.

^c^CGI improved: 1–2 points, CGI-I no change: 3–5 points, CGI-I worsened: 6–7 points. Δ-CPRS-M improved: ≥2 points decrease, Δ-CPRS-M no change: ≤±1 points’ change, Δ-CPRS-M worsened: ≥2 points increase.

ECT = Electroconvulsive Therapy, Q1 = first quartile, Q3 = third quartile, CGI-I = Clinical Global Impression - Improvement Scale, EQ-VAS = EuroQol Visual Analogue Scale, CPRS-M = memory item from the Comprehensive Psychopathological Rating Scale.

### Outcomes

Median ALLO and PROG concentrations before and after the first ECT seizure are shown in Table 2. In the total cohort, the median concentrations did not change after the first ECT seizure (ALLO *p* = 0.16, PROG *p* = 0.22). When data were stratified by sex, ALLO concentrations did not change in either group, but postictal PROG increased in men (33 increased, 17 decreased, *p* = 0.03).

**Table 2:** Neuroactive steroid concentrations before and after ECT seizure compared with the sign test

		Before		After		
	*n*	median	Q1,Q3	median	Q1,Q3	*p*
ALLO, ng/mL						
Whole cohort	130	3.63	2.49, 7.36	4.17	2.75, 8.04	0.16
Women	80	4.66	2.86, 10.16	4.45	2.76, 8.43	0.50
Men	50	2.96	2.11, 4.34	3.54	2.72, 6.20	0.20
PROG, pg/mL						
Whole cohort	130	40.75	21.00, 78.38	44.00	25.38, 75.25	0.22
Women	80	49.75	24.00, 86.13	43.75	25.13, 82.13	1.00
Men	50	29.25	15.50, 63.13	44.00	26.13, 64.00	0.03

ECT: electroconvulsive therapy. ALLO: allopregnanolone. PROG: progesterone.

**Fig. 1:**
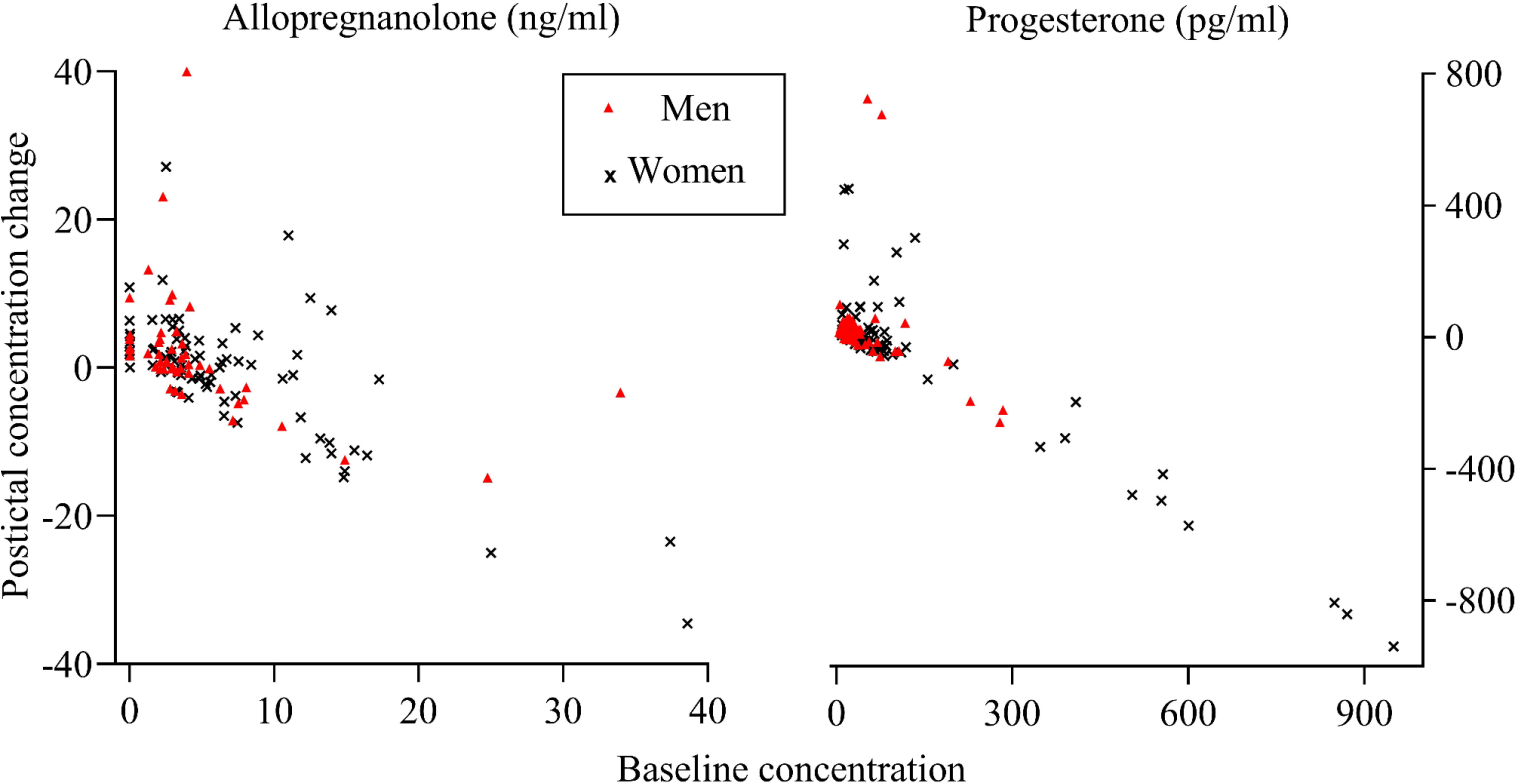
Scatter plots of postictal concentration change after the first ECT seizure by baseline concentration. The relation of baseline concentrations and postictal concentration change for allopregnanolone (left) and progesterone (right)

Baseline ALLO and PROG were higher in women than in men (both *p* < 0.01). In women, only the postictal ALLO and PROG concentrations were positively correlated (women, *r* = 0.33, *p* = 0.03; men, *r* = 0.13, *p* = 0.38), as were the Δ-ALLO and Δ-PROG concentrations (women, *r* = 0.30, *p* = 0.006; men, *r* = 0.01, *p* = 0.94). The concentration change correlated negatively with the baseline concentration for both ALLO (*r*=-0.61, *p* < 0.001) and PROG (*r*=-0.60, *p* < 0.001), as shown in Fig. 1. This negative correlation remained when the data were stratified by sex (both *p* < 0.001).

Two outliers are excluded for better visualization. Outlier 1 (female): allopregnanolone baseline 115, postictal change − 108. Progesterone baseline 2204, postictal change − 2182. Outlier 2 (male): allopregnanolone baseline 2204, postictal change − 2182. Progesterone baseline 1692, postictal change − 2182.

There was no correlation between Δ/baseline-ALLO or Δ/baseline-PROG and peak HR (ALLO *r*=-0.00, *p* = 0.98; PROG *r*=-0.02, *p* = 0.98), EEG seizure duration (ALLO *r*=-0.07, *p* = 0.43; PROG *r* = 0.01, *p* = 0.94), or Δ/baseline prolactin (ALLO *r*=-0.09, *p* = 0.43; PROG *r*=-0.04, *p* = 0.75). When correlations were stratified by sex, Δ/baseline-ALLO and EEG duration correlated negatively in men (*r*=-0.33, *p* = 0.02), but there were no other significant correlations (all *p* > 0.26, data not shown).

Regression analyses revealed no impact of ALLO or PROG concentrations (baseline or Δ –concentrations) on outcome at the completed ECT series as rated on CGI-I, CPRS-M, or EQ-VAS (as shown in Tables 3 and 4).

**Table 3:** Logistic binary regressions investigating neuroactive steroid concentrations at first seizure as predictors of clinical outcome

	CGI-I (*n* = 117)		CPRS-M (*n* = 85)	
	OR	95% CI	OR	95% CI
Concentrations before seizure				
Allopregnanolone, ng/mL	0.99	(0.97–1.02)	0.95	(0.86–1.05)
Progesterone, pg/mL	1.00	(1.00–1.00)	1.00	(1.00–1.00)
Concentration change after seizure				
Allopregnanolone, ng/mL	1.00	(0.97–1.03)	1.03	(0.96–1.10)
Progesterone, pg/mL	1.00	(1.00–1.00)	1.00	(1.00–1.00)

CGI-I = Clinical global impressions improvement scale, CPRS-M = memory item from the Comprehensive Psychopathological Rating Scale, ECT = Electroconvulsive therapy, OR = odds ratio, CI = confidence interval.

Clinical outcome is defined as improvement (1 or 2 points) on the CGI-I scale and impairment (minimum 2 points increase) on the CPRS-M scale after the completed ECT series.

**Table 4:** Simple linear regressions investigating neuroactive steroid concentrations at first seizure as predictors of clinical outcome

	EQ-VAS (*n* = 81)		
	*b*	*SE*	*p*
Concentrations before seizure			
Allopregnanolone, ng/mL	-0.12	0.19	0.55
Progesterone, pg/mL	-0.01	0.01	0.40
Concentration change after seizure			
Allopregnanolone, ng/mL	0.12	0.19	0.54
Progesterone, pg/mL	0.01	0.01	0.35

EQ-VAS = EuroQol visual analogue scale, ECT = electroconvulsive therapy, b = unstandardized b-coefficient, SE = standard error.

Outcome variable is change in EQ-VAS rating after the completed ECT series.

In the group with data available in the medical charts on postictal suppression (*n* = 62), participants with postictal suppression had higher median baseline concentrations of both ALLO (4.80 versus 2.18 ng/mL, *p* = 0.01) and PROG (32.00 versus 17.00 pg/mL, *p* = 0.02). The groups did not differ in Δ/baseline or postictal ALLO or PROG concentrations (all *p* > 0.18, data not shown).

### Post-hoc analyses

Although the majority of participants were referred for ECT with the indication depression, the group was heterogeneous, including both uni- and bipolar depression as well as depressive episodes with and without psychosis. Post-hoc analyses were therefore performed to explore potential differences in hormone regulation between subtypes of depression. Baseline concentrations of ALLO or PROG did not differ between unipolar versus bipolar depression (ALLO *p* = 0.82; PROG *p* = 0.10), nor between depression with and without psychotic symptoms (ALLO *p* = 0.78; PROG *p* = 0.91). Repeating the primary analysis in the four subgroups showed no change in median ALLO before and after the ECT seizure (all *p* ≥ 0.11, data not shown). PROG concentration change in the unipolar subgroup was at the border of significance, with 57 increasing and 37 decreasing (median (Q1, Q3) PROG before 31.75 (18.88, 78.63) pg/mL; after 47.00 (26.50, 76.50) pg/mL, *p* = 0.05). There was no change in median PROG in the other three subgroups (all *p* ≥ 0.26, data not shown). Median PROG change did not differ between sexes in the unipolar group (*p* = 0.06).

## Discussion

The first epileptic seizure of an ECT series did not affect postictal concentrations of ALLO or PROG in the total cohort, but there was a significant increase in median PROG concentrations in the male subgroup and a borderline significant increase in the subgroup with unipolar depression. In men, the increase in postictal ALLO correlated negatively with EEG seizure duration. Postictal EEG suppression was associated with higher baseline ALLO and PROG concentrations. Apart from that, ALLO or PROG concentrations did not correlate with seizure generalization parameters or clinical outcome rating scales.

### Interpretation and comparison with previous findings

Case series of postictal ALLO concentrations in children [25] and fertile women in the late follicular phase [24] reported postictal increases in ALLO but not in PROG [24, 25]. Our findings of unchanged postictal ALLO concentrations imply that a seizure induced during ECT does not cause changes in ALLO concentrations in peripheral blood similar to those observed in spontaneous epileptic seizures. This could reflect differences in seizure generalization, or less physiological stress during ECT due to supportive actions, e.g., preoxygenation and muscle relaxants.

In contrast to epileptic seizures in fertile women [24], we found an increase in postictal PROG in men. We have not found any previous studies on PROG in men after epileptic seizures. Possible state-dependent changes in PROG in men with severe psychiatric illness are still poorly understood [47], but a recent study reported lower serum concentrations of both ALLO and PROG in men with bipolar disorder compared to healthy controls [48]. It is possible that lower baseline concentrations in men allow for higher sensitivity in detecting postictal changes. In men and rodents, acute stress induces release of PROG along with cortisol from the adrenal glands by activation of the hypothalamic-pituitary-adrenal axis [49–51]. In women, findings are more inconsistent, possibly due to altered HPA reactivity in different phases of the menstrual cycle, or to comparatively higher gonadal PROG in the luteal phase affecting results [52–54]. Transient increases in cortisol after ECT is also a consistent finding [36, 55–57]. The increase in postictal PROG observed in men might thus reflect either a discrete physiologic effect of epileptic seizures or adrenal PROG release due to physiologic stress during ECT treatment, although these speculations should be empirically tested in future studies.

Our finding of increased PROG in the subgroup with unipolar depression was not part of the pre-planned analyses, and results were just at the border of significance. Considering the multiple statistical tests included in the post-hoc analysis, a spurious false positive result cannot be ruled out. Contrary to men [48], a previous study in women with stable affective disorder reported higher concentrations of both ALLO and PROG in the luteal phase than controls, most pronounced in bipolar disorder [58]. Possibly, bipolar disorder entails decreased adrenal release of PROG but higher reactivity in cyclic gonadal release and our study results could be interpreted as a result of reduced adrenal gland response in bipolar versus unipolar depression.

ALLO and PROG postictal changes correlated negatively with baseline concentrations, both in the whole study cohort and in subgroups divided by sex, possibly reflecting normalization from baseline levels, if not a statistical effect of regression to the mean. This could imply that ECT seizures affect neuroactive steroids differently in a state-dependent manner and might reflect the heterogeneity in our study cohort compared to the patient samples included in the previous reports of ALLO and PROG in epileptic seizures [24, 25]. Repeated sampling and a control group not undergoing ECT should ideally have been included to assess whether the normalization of hormonal values was affected by ECT.

We found no correlation between baseline neuroactive steroid concentrations and seizure generalization markers in the total cohort, which suggests that physiological concentrations of ALLO or PROG have no major effect on ECT seizure propagation. Contrary to our hypothesis, the increase in postictal ALLO correlated negatively with EEG seizure duration in men. Considering the probability of poorly generalized seizures in our cohort (as discussed under ***Limitations***), this negative correlation could reflect that shorter seizures were associated with a greater degree of generalization and more pronounced postictal GABAergic inhibition [59]. If so, this might be more easily detected in the male subgroup due to lower baseline ALLO concentrations. However, multiple comparisons and heterogeneous data warrant cautious interpretation of these findings, considering the lack of correlation between neuroactive steroid concentrations and other generalization parameters. The positive correlation between postictal EEG suppression and baseline levels of ALLO and PROG must be interpreted even more cautiously due to the amount of missing data. If our preliminary finding of a correlation between ALLO and PROG serum concentrations and postictal EEG suppression would be replicated it might reflect the involvement of ALLO and PROG in increased GABAergic inhibition during the postictal phase [59]. Another tentative speculation is that neuroactive steroids affect the efficacy of intrinsic mechanisms of seizure termination, considering previous links between ALLO/PROG and seizure activity in epilepsy [60].

### Limitations

The timing of the blood samples is an important limitation; only one postictal sample was collected, and other than the specification ‘within 30 minutes from seizure termination’, the interval between seizure termination and blood sampling was not recorded. Previous reports found a marked increase in postictal ALLO at 15 min [24] and 30 min [25] after seizure termination. Literature on postictal PROG is scarce, but animal studies on stress-induced effects have reported more rapid normalization (peaking at 10 min and normalization at 30 min) [61]. Postictal prolactin after ECT seizures peaks at approximately 10–15 min after seizure termination [57]. Thus, our samples might have been drawn after postictal PROG and prolactin had peaked, resulting in underestimation of postictal concentrations. As postictal ALLO has a slower increase and normalization, the impact of imprecise timing is likely lessened, but maximum postictal concentrations might not be detected. Using rank comparisons in statistical calculations rather than parametric tests might, however, mitigate the risk of underestimating possible correlations.

Previous studies on ALLO and PROG directly after seizures included more homogenous populations than our study; 11 patients aged 3–8 years [25] and 10 seizures among seven fertile women during the late follicular phase of the menstrual cycle [24]. Serum concentrations of ALLO and PROG are lower in childhood than after puberty onset [62], and during the menstrual cycle of fertile women, the highest serum concentrations of ALLO are reported in the late luteal phase [63]. Our study cohort included fertile women with no control of the menstrual cycle or use of hormonal contraceptives. This would increase variability in baseline levels of ALLO and PROG and possibly make it more difficult to detect a relative postictal concentration increase in the female subgroup if a large part of circulating ALLO and PROG originate from the gonads or medication.

This study lacks a control group for comparison of effects of pharmacological effects on ALLO and PROG concentrations without a concurrent ECT seizure. Therefore we cannot with discriminate seizure effects from potential effects of anaesthetics and muscle relaxants. Another factor to consider is the exclusive right unilateral electrode placement in this cohort, which carries a higher risk of poor seizure generalization than bilateral electrode placement [64]. This is further implied by the relatively low peak HR of 120 (*SD* = 24) compared to previous reports of mean peak HR in effective ECT seizures [65]. Thus, our cohort might contain a relatively large proportion of patients with poorly generalized seizures.

The data on EEG suppression suffered a large amount of missing data (68 of 130), as this assessment was omitted in many charts and thus could not be retrieved retrospectively.

## Conclusion

In this first study of ALLO and PROG concentrations before and after the first seizure in an ECT series, we found no postictal changes. Nor were there any correlations between ALLO or PROG and seizure generalization parameters or clinical outcome ratings at the end of the full ECT series.

However, in men, postictal PROG increased, which might be due to lower baseline levels and less gonadal PROG release, allowing for higher sensitivity. PROG in men during psychiatric illness and ECT could be worth exploring further in a prospective study design. Our finding that higher baseline ALLO and PROG are associated with postictal EEG suppression should be considered preliminary until replicated.

## Data Availability

Due to institutional and ethical constraints, we have restrictions regarding the processing of personal data. The research participants did not consent to public sharing of pseudonymized data, and thus, we do not have ethical permission to openly share these data. Neither do we have a legal permission according to the General Data Protection Regulation (GDPR) regarding classified personal data.

## Abbreviations

ALLO: Allopregnanolone
CGI-I: The Clinical Global Impression-Improvement Scale
CPRS-M: The memory item from the Comprehensive Psychopathological Rating Scale
ECT: Electroconvulsive therapy
EEG: Electroencephalography
EQ-VAS: The Euro-Qol Visual Analogue Rating Scale
GABA: γ-aminobutyric acid
HR: Heart Rate
NPX: Normalized protein expression
PREFECT: Predictors For ECT multicentre study
PROG: Progesterone
Q1: First quartile
Q3: Third quartile
Q-ECT: The Swedish National Quality Register for ECT
SD: Standard deviation
